# Phenotypic and genotypic detection of carbapenemase-producing *Escherichia coli* and *Klebsiella pneumoniae* in Accra, Ghana

**DOI:** 10.1371/journal.pone.0279715

**Published:** 2022-12-30

**Authors:** Felicia P. Dwomoh, Fleischer C. N. Kotey, Nicholas T. K. D. Dayie, Mary-Magdalene Osei, Felicia Amoa-Owusu, Vida Bannah, Fuad M. Alzahrani, Ibrahim F. Halawani, Khalid J. Alzahrani, Beverly Egyir, Eric S. Donkor

**Affiliations:** 1 Department of Medical Microbiology, University of Ghana Medical School, Korle Bu, Accra, Ghana; 2 Department of Medical Laboratory, University of Ghana Medical Centre, Legon, Accra, Ghana; 3 FleRhoLife Research Consult, Teshie, Accra, Ghana; 4 Department of Bacteriology, Noguchi Memorial Institute for Medical Research, University of Ghana, Legon, Accra, Ghana; 5 Department of Clinical Laboratory Sciences, College of Applied Medical Sciences, Taif University, Taif, Saudi Arabia; Cornell University, UNITED STATES

## Abstract

**Aim:**

To describe the occurrence of carbapenem resistance among multidrug-resistant (MDR) *Escherichia coli* and *Klebsiella pneumoniae* isolated from clinical specimens in Accra using phenotypic and genotypic methods.

**Methodology:**

The study was cross-sectional, involving 144 clinical MDR *E*. *coli* and *K*. *pneumoniae* isolates recovered from the Central Laboratory of the Korle Bu Teaching Hospital (KBTH). The isolates were re-cultured bacteriologically, identified using standard biochemical tests, and subjected to antibiotic susceptibility testing using the Kirby-Bauer method. Carbapenem resistance was determined based on imipenem, meropenem, and ertapenem zones of inhibition, as well as minimum inhibitory concentrations (MICs). Carbapenemase production was determined phenotypically by modified Hodge test (MHT) and modified carbapenem inactivation method (mCIM), and genotypically with multiplex PCR targeting the *blaKPC*, *blaIMP*, *blaNDM*, *blaVIM*, and *blaOXA*-48 genes.

**Results:**

Of the 144 MDR isolates, 69.4% were *E*. *coli*, and 30.6% were *K*. *pneumoniae*. The distribution of antimicrobial resistance rates among them was ampicillin (97.2%), cefuroxime (93.1%), sulfamethoxazole-trimethoprim (86.8%), tetracycline (85.4%), cefotaxime and cefpodoxime (77.1% each), amoxicillin-clavulanate (75%), ceftriaxone (73.6%), ciprofloxacin (70.8%), levofloxacin (66.0%), cefepime (65.3%), ceftazidime (64.6%), gentamicin (48.6), piperacillin-tazobactam (40.3%), cefoxitin (14.6%), amikacin (13.9%), ertapenem and meropenem (5.6% each), and imipenem (2.8%). In total, 5.6% (8/144) of them were carbapenem-resistant (carbapenem MIC range = 0.094–32.0 μg/ml), with 75% (6/8) of these testing positive by the phenotypic tests and 62.5% (5/8) by the genotypic test (of which 80% [4/5] carried *blaOXA*-48 and 20% (1/5) *blaNDM*). The *blaVIM*, *blaIMP*, and *blaKPC* genes were not detected.

**Conclusion:**

Although the rates of antibiotic resistance among the isolates were high, the prevalence of carbapenemase producers was low. The finding of *blaOXA*-48 and *blaNDM* warrants upscaling of antimicrobial resistance surveillance programmes and fortification of infection prevention and control programmes in the country.

## Introduction

*Escherichia coli* (*E*. *coli*) and *Klebsiella pneumoniae* (*K*. *pneumoniae*) are two of the major gut-colonizing Gram-negative bacilli of the Enterobacterales implicated in meningitis, infections of the gastrointestinal and urinary tracts, septicaemia, and other infections, most of which are multidrug resistant (MDR) [1–5]. Such resistance traits have a global distribution, and threaten the efficacy of antibiotics [6–9]. In the last few years, there has been an increase in the pervasiveness of multidrug resistance in the Enterobacterales, primarily mediated by the acquisition of resistance trait-encoding genes, such as those that code for extended-spectrum beta-lactamase (ESBL) [10–14]. Carbapenems, such as imipenem, meropenem, and ertapenem, have been used as the last resort in the treatment of such infections, but resistance to them has emerged and is spreading fast [15–22]. Carbapenem resistance is mainly mediated by carbapenemase-encoding genes, the major types of which are Class A *Klebsiella pneumoniae* carbapenemases (*blaKPC*), Class B Metallo-β-lactamases (*blaNDM*, *blaVIM*, and *blaIMP*), and Class D OXA β-lactamases (*blaOXA*); mobile genetic elements (transposons and plasmids) facilitate their transmission [17]. Carbapenem resistance can also occur due to porin deficiencies, which leads to a reduction in the entry of ß-lactam drugs into bacteria via their cell membranes [23].

Aptly, antimicrobial resistance (AMR) has been classified as a major threat to modern medicine by the World Health Organization (WHO), with carbapenemase-producing Enterobacterales (CPE) and carbapenemase-resistant Enterobacterales (CRE) compositely identified as a growing global menace [24]. About fifty thousand lives are lost annually in Europe and the US alone due to infections caused by resistant pathogens [25]. It is projected that failure to adequately deal with this menace could lead to an annual death of 10 million people, a 2–3.5% reduction in gross domestic product, and an overall cost of US$ 100 trillion by the year 2050 [26]. This AMR burden is expected to be somewhat higher in sub-Saharan Africa with a decline in a gross domestic product of $2,895 billion, representing 20% of the region’s total economic output [26]. In 2017, the WHO cited CRE as being among the highest critical category of the global priority list of pathogens, probably because carbapenems are considered as part of the limited last-line antimicrobials [27]. The rapid spread of CRE and CPE amid the slow-paced discovery of newer antimicrobials have made CRE and CPE a significant public health problem around the world [16]. They remain a part of the most difficult-to-treat MDR infections globally [28], and their infections are associated with high morbidity and mortality rates ranging from 30% to 75% [18] and increased healthcare costs ranging from USD 22,484 to USD 66,031 per patient in the USA [19].

Studies across different geographical areas have reported increasing prevalence of CRE/CPE with endemic places being the USA, India, and Greece, and an imminent worldwide CPE epidemic has been predicted [6]. In Italy, there was a rise of 1.3% to 15.2% prevalence of carbapenem-resistant *K*. *pneumoniae* from 2006 to 2010, and Hungary recorded a rise from 0.0% to 5.5% during the same period [29]. Additionally, data from the National Healthcare Safety Network (NHSN) showed that between the period 2000 and 2008, there was a significant rise in MDR *K*. *pneumoniae* and *E*. *coli* (from 7.0% to 13%) in the United States [29]. A systematic review by Manenzhe *et al*. [30] in Africa highlighted increasing reports of carbapenemase-producing bacteria in hospital environs—from 2.3% to 67.7% in North Africa and 9% to 60% in sub-Saharan Africa. In Ghana, a study conducted by Owusu-Oduro [31] reported the prevalence of CRE in urinary tract infection (UTI) patients to be 10.0%. Additionally, Hackman *et al*. [32] in Accra also established the emergence of CRE, reporting a prevalence of 7.2% among phenotypic ESBL isolates. To add to this, a study conducted by Codjoe *et al*. [33] reported a carbapenem resistance prevalence of 2.9%, with even non-lactose fermenters contributing significantly to this proportion.

The high clinical significance of CRE/CPE and the immense global health threat they pose calls for active CRE/CPE surveillance in different parts of the world. As the organisms most implicated in CRE/CPE infections are *K*. *pneumoniae* and *E*. *coli* [8, 34], surveillance of carbapenem-resistant *K*. *pneumoniae* and *E*. *coli* infections is an important strategy in combating the public health threat posed by CRE and other AMR pathogens globally. However, very few studies have been conducted on these infections in Ghana and other parts of Africa. The first-ever study on the detection and characterization of carbapenem resistance genes in Ghana focused mostly on *Pseudomonas aeruginosa* and *Acinetobacter baumannii* and the various carbapenemase enzymes associated with them [35]. Owusu-Oduro [31] and Hackman *et al*. [32], who also contributed data on carbapenem-resistant isolates in Ghana, limited their detection to disc diffusion and minimum inhibitory concentration (MIC) determination with E-Test strips, with no molecular characterizations done. Codjoe *et al*. [33] addressed this limitation, but studied a wide range of Gram-negative bacteria, and just a few of these were *K*. *pneumoniae*. Besides these, detecting and distinguishing between carbapenemases is not routinely done by diagnostic laboratories in the country, a phenomenon that pertains to most resource-poor settings [36]. Hence there are several gaps in knowledge on carbapenem resistance in *E*. *coli* and *K*. *pneumoniae* in the country, particularly, on harboured carbapenemase genes. This information is crucial for effective antimicrobial therapy and infection prevention and control. To help fill the identified knowledge gaps, this study evaluated the prevalence of carbapenem resistance, AMR patterns, and the distribution of carbapenem resistance genes among MDR *E*. *coli* and *K*. *pneumoniae* isolated from clinical specimens in Accra, Ghana.

## Materials and methods

### Study site, design, and sampling

This study was carried out at the Central Microbiology Laboratory of the Korle Bu Teaching Hospital (KBTH), and received approval from the Ethical and Protocol Review Committee of the College of Health Sciences, University of Ghana (Protocol Identification Number: CHS-Et/M.7—5.2 / 2020–2021) and the Institutional Review Board of KBTH (Protocol Identification Number: STC/IRB/000121/2020). The study was cross-sectional, involving 144 clinical, non-duplicate MDR *E*. *coli* and *K*. *pneumoniae* isolates that were randomly selected from wound, urine, sputum, blood, and miscellaneous (pus, pleural fluid, aspirate, ear, eye and high vaginal swab) samples, and processed by the Central Microbiology Laboratory of KBTH from December 2020 to March 2021. In this study, MDR was defined as isolates resistant to at least one agent belonging to three or more antibiotic classes [8]. Patients’ information on clinical diagnosis and demographics were obtained from de-identified patient records using a clinical data collection form. These data collection forms were completed and accompanied each bacterial isolate that was collected. The isolates were collected into tryptic soy broth (TSB) (Oxoid, Basingstoke Hampshire, UK) containing 10% glycerol, and within four hours of collection, conveyed to the Department of Medical Microbiology, University of Ghana Medical School, for laboratory processing. These isolates were stored under -20 °C until further analysis.

### Laboratory analysis

Stored clinical isolates were retrieved from the freezer (-20 °C), allowed to thaw, vortexed, and re-cultured on MacConkey agar and blood agar plates (Beckton, Dickinson and Company, USA). These agar plates were then incubated aerobically at 37 °C for 18–24 hours. A presumptive diagnosis was done based on colonial morphology and Gram staining, while identification was based on appropriate conventional biochemical tests. The MDR status of *E*. *coli* and *K*. *pneumoniae* was further confirmed by performing antibiotic susceptibility testing (AST) using the Kirby-Bauer disc diffusion method on Mueller-Hinton agar (MHA) plates (Oxoid, Basingstoke Hampshire, UK), and zones of inhibition compared to that of the CLSI [37] guidelines for interpretation. The following antibiotics were used: meropenem (10 μg), ertapenem (10 μg), imipenem (10 μg), piperacillin-tazobactam (100/10 μg), ceftriaxone (30 μg), cefuroxime (30 μg), sulfamethoxazole-trimethoprim (1.25/23.75 μg), cefpodoxime (10 μg), gentamicin (10 μg), cefoxitin (30 μg), amoxiclav (20/10 μg), ampicillin (10 μg), cefotaxime (30 μg), ceftazidime (30 μg), levofloxacin (5 μg), ciprofloxacin (5 μg), amikacin (30 μg), cefepime (30 μg) and tetracycline (30 μg) (Oxoid, Basingstoke Hampshire, UK; Mast Group, UK). Carbapenem resistance was defined based on resistance to any or all of ertapenem, imipenem, and meropenem, using the cut-offs of <22 mm (for ertapenem), <23 mm (for imipenem), and <23 mm (for meropenem) specified in the CLSI [37] guidelines.

Confirmation of disc diffusion-based resistance profiles of carbapenems was performed using the E-test method. This was done by preparing a suspension of the *E*. *coli* and *K*. *pneumoniae* isolates using sterile 0.85% saline. These colony suspensions were each adjusted to 0.5 McFarland standard turbidity and inoculated onto MHA (Oxoid, Basingstoke Hampshire, UK) before the antibiotic-impregnated E-test strips were applied. An inhibition ellipse centered along the strip was formed after 16–18 hours of incubation at 35 °C ± 2 °C ambient air. At the point where the inhibition ellipse edge intersects with the strip, the minimum inhibitory concentration (MIC) was read directly from the scale in micrograms per millilitre (μg/ml). MICs using E-tests were performed with these breakpoints from CLSI [37]: ertapenem (≤ 0.5 μg/ml), imipenem (≤1 μg/ml) and meropenem (≤1 μg/ml) [Enterobacteriaceae that produce carbapenemases usually test intermediate or resistant to one or more carbapenems].

Additional phenotypic confirmatory tests, both modified carbapenem inactivation method (mCIM) and modified Hodge test (MHT), were also performed, as previously described [37, 38]. Briefly, in the mCIM test, a sterile bacteriological loop was used to pick 1 μL loopful each of isolates of *K*. *pneumoniae* and *E*. *coli* from overnight blood agar (Oxoid, Basingstoke Hampshire, UK) cultures and separately emulsified in 2mL TSB (Oxoid, Basingstoke Hampshire, UK) in test tubes. The inoculums were then vortexed, and a 10 μg meropenem disc was added to each test tube. The TSB-meropenem disc suspension in the test tubes was incubated at 37 °C for four hours. Following the incubation for 4 hours, a 0.5 McFarland suspension of *E*. *coli* ATCC 25922 was prepared and inoculated onto MHA plates (Oxoid, Basingstoke Hampshire, UK). The meropenem discs were removed from each TSB-meropenem disc suspension using a 10 μL loop and then placed on the MHA plate previously seeded with *E*. *coli* ATCC 25922. These plates were then incubated at 37 °C for 18–24 hours. Following incubation, zones of inhibition were measured, and isolates with zone diameters between 6–15 mm (or the presence of pinpoint colonies within 16–18 mm zones) were considered carbapenemase-positive. Regarding the MHT, it involved a 1 in 10 dilution of a 0.5 McFarland standard suspension of *E*. *coli* ATCC 25922 seeded on MHA (Oxoid, Basingstoke Hampshire, UK) plates. Ertapenem discs (10 μg) were placed at the centre of each of these seeded plates, after which the test isolates (*K*. *pneumoniae* and *E*. *coli* cultured on blood agar for 18 to 24 hours) were streaked from the edge of the plate to the edge of the disc in a straight line. Afterwards, all the plates were incubated at 37 °C for 18 to 22 hours. Strains that showed a cloverleaf shape around the ertapenem discs were considered positive for carbapenemase activity. *K*. *pneumoniae* ATCC BAA-1705 and *E*. *coli* ATCC 25922 served as positive and negative control strains, respectively.

Molecular detection of carbapenem resistance genes was performed at the Bacteriology Department, Noguchi Memorial Institute for Medical Research. Crude DNA was obtained from isolates using the boiling method and used as a template for PCR. Each reaction mix (25 μL) contained 12.5 uL of 2 x DreamTaq Green PCR Master Mix, 4.5 μL of primer mix, 6 μL of molecular grade nuclease-free water, and 2 μL of DNA template. For detection of carbapenemase enzymes, 5 primers were chosen from the 3 classes of carbapenemase genes: Class A (KPC gene), Class B meta-llo-beta-lactamases (NDM, VIM, IMP), and Class D oxacillinases (OXA-48) [39]. *K*. *pneumoniae* ATCC BAA 1705 was used as a positive control. Amplification was done at 94 °C for 3 minutes as the initial step for denaturation, followed by 35 cycles of denaturation at 94 °C for 30 seconds, annealing at 61.6 °C for 30 seconds and extension at 72 °C for 1 minute. Final elongation was at 72 °C for 7 minutes. All PCR amplicons were analyzed by horizontal gel-electrophoresis in a 2% (weight/volume) (SeaKem^®^GTG^®^Agarose, Lonza) Tris/Acetate/EDTA 50 x concentrate buffer. The agarose was stained with 5 uL of Gel-red (Bio-Rad, UK). About 5 μL of amplicons were put into the wells and run in a 1 x Tris-acetate EDTA (TAE) at 100 volts for 1 hour. A 100 bp molecular ladder (Fermentas, Germany), was used as a marker. The amplicons were visualized with the Ultra-violet Gel Illuminator (UVP BioDoc-It^2^ Imager).

Details of the primer sequences that were used in the detection of the carbapenemase-producing isolates are presented in Table 1.

**Table 1:** Primer sequences used in the detection of the carbapenemase-producing isolates.

Carbepenemase Genes	Amplicon size (bp)	Primer Sequence (5’—3’)	References
*bla*_*KPC*_	683	FP: GTATCGCCGTCTAGTTCTGC	Obeng-Nkrumah *et al*. [14]
		RP: 5’-GGTCGTGTTTCCCTTTAGCC	
*bla*_VIM_	390	FP: GATGGTGTTTGGTCGCATA	Poirel *et al*. [40]
		RP: CGAATGCGCAGCACCAG	
*bla*_IMP_	188	FP: GGAATAGAGTGGCTTAAYTCTC	Obeng-Nkrumah *et al*. [14]
		RP: CCAAACYACTASGTTATCT	
*bla*_*OXA-48*_	438	FP: GCGTGGTTAAGGATGAACAC	Poirel *et al*. [40]
		RP: CATCAAGTTCAACCCAACCG	
*bla*_*NDM*_	760	FP: GAAGCTGAGCACCGCATTAG	Obeng-Nkrumah *et al*. [14]
		RP: TGCGGGCCGTATGAGTGATT	

FP = forward primer; RP = reverse primer; *bla*_*KPC*_ = *Klebsiella pneumoniae* carbapenemase; *bla*_VIM_ = Verona Intergron-encoded Metallo-β-Lactamases; *bla*_IMP_ = Imipenem-resistant Pseudomonas; *bla*_*OXA-48*_ = Oxacillinase—48; *bla*_*NDM*_ = New Delhi Metallo-β-Lactamases

### Data analysis

Statistical Products and Services Solutions (SPSS), an Excel-based statistical tool, was used to enter and analyse the data. Descriptive analyses such as counts, frequencies, and percentages were computed. Proportions were compared using the Chi-square test. The prevalence was calculated by dividing the number of occurrences of carbapenem-resistant *E*. *coli* and *K*. *pneumoniae* strains by the total number of MDR *E*. *coli* and *K*. *pneumoniae* collected during the study period, and this was stratified based on gender. P values less than or equal to 0.05 were interpreted as statistically significant.

### Ethical approval

This study received approval from the Ethical and Protocol Review Committee of the College of Health Sciences, University of Ghana, with Protocol Identification Number: CHS-Et/M.7—5.2 / 2020–2021 and the Institutional Review Board of KBTH, with Protocol Identification Number: STC/IRB/000121/2020.

## Results

### Demographics and characteristics of the bacterial isolates

A total of one hundred and forty-four (144) *E*. *coli* and *K*. *pneumoniae* isolates were evaluated in this study. Among these, 69.4% (*n* = 100) were *E*. *coli*, and 30.6% (*n* = 44) were *K*. *pneumoniae*. The study isolates were collected from fifteen (15) different sample types, with the majority of them isolated from urine (68.75%), followed by wound (9.0%), blood (4.9%), and other sources (Table 2). Regarding the gender distribution of the participants from whom the isolates were recovered, 59.0% (*n* = 85) were females and 41.0% (*n* = 59) were males (Table 2). The study participants were aged between 3 days and 89 years, and the overall mean age was 41.14 ± 24.5 years; the mean age for males was 49.10 ± 27.72 years, and that of females was 35.61 ± 20.41 years. Most of the isolates were from patients above the age of 60 years (25.7%), followed by the age range of 31 to 40 years (20.1%), with the least number of samples collected from those within the age range of 11 to 20 years (5.6%).

**Table 2:** Distribution of bacterial isolates by gender, age, and sources of sample collection.

Demographic Characteristics	Bacterial Isolates				
	*E*. *coli*		*K*. *pneumoniae*		Total (*n*, %)
	*n*	%	*n*	%	
***Gender***					
Female	58	40.3	27	18.8	85 (59.0)
Male	42	29.2	17	11.8	59 (41.0)
***Age (years)***					
≤ 10	12	8.3	9	6.3	21 (14.6)
11–20	4	2.8	4	2.8	8 (5.6)
21–30	15	10.4	6	4.2	21 (14.6)
31–40	23	16.0	6	4.2	29 (20.1)
41–50	8	5.6	6	4.2	14 (9.7)
51–60	9	6.3	5	3.5	14 (9.7)
> 60	29	20.1	8	5.6	37 (25.7)
***Type of sample***					
Abscess	1	0.7	0	0.0	1 (0.7)
Aspirate	3	2.1	1	0.7	4 (2.8)
Blood	4	2.8	3	2.1	7 (4.9)
Catheter tip	1	0.7	0	0.0	1 (0.7)
Cord	0	0.0	1	0.7	1 (0.7)
Ear	1	0.7	3	2.1	4 (2.8)
Endocervix	3	2.1	0	0.0	3 (2.1)
Vagina	5	3.5	1	0.7	6 (4.2)
Nose	1	0.7	0	0.0	1 (0.7)
Pleural fluid	0	0.0	1	0.7	1 (0.7)
Sputum	0	0.0	1	0.7	1 (0.7)
Throat	0	0.0	1	0.7	1 (0.7)
Urethral	1	0.7	0	0.0	1 (0.7)
Urine	70	48.6	29	20.1	99 (68.8)
Wound	10	6.9	3	2.1	13 (9.0)

### Resistance patterns of the bacterial isolates

**Fig 1:**
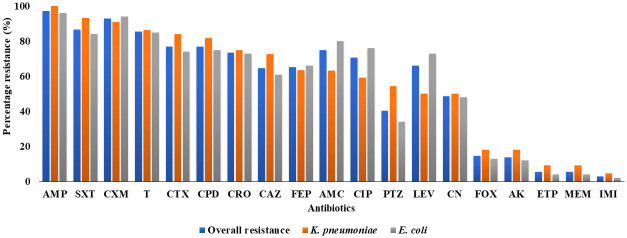
Antimicrobial resistance patterns of the study isolates. In the figure, MEM = Meropenem; IMI = Imipenem; ETP = Ertapenem, PTZ = Piperacillin/tazobactam; AMC = Amoxicillin/clavulanate; AMP = Ampicillin; CRO = Ceftriaxone; CXM = Cefuroxime; CPD = Cefpodoxime; FOX = Cefoxitin; CTX = Cefotaxime; FEP = Cefepime; CAZ = Ceftazidime; LEV = Levofloxacin; CIP = Ciprofloxacin; AK = Amikacin; CN = Gentamicin; T = Tetracycline; SXT = Sulfamethoxazole-trimethoprim.

Resistance to ampicillin (97.2%), cefuroxime (93.1%), and imipenem (2.8%) were observed among the isolates. Therefore, the overall prevalence of carbapenem resistance among *E*. *coli* and *K*. *pneumoniae* according to the Kirby-Bauer disc diffusion method was 5.6%. Among the eight isolates, 50% (*n* = 4) were *E*. *coli*, and the other half were *K*. *pneumoniae*. All the eight carbapenem-resistant isolates showed 100% resistance to both meropenem and ertapenem; 50% of them were resistant to imipenem. Fig 1 shows antibiotic resistance patterns (disc diffusion method) of the study isolates.

In this study, carbapenem MICs ranged from 0.094 μg/ml to 32.0 μg/ml. Ertapenem MIC (which demonstrated two isolates to be carbapenem-resistant and one to be intermediate carbapenem-resistant) seemed to better predict carbapenemase gene presence compared to those of imipenem and meropenem (each of which showed the five carbapenemase gene-harbouring isolates to be carbapenem-sensitive). Also, all the five carbapenemase gene-harbouring isolates were both ertapenem- and meropenem-resistant. Overall, MIC values for isolates with carbapenemase-encoding genes were lower in comparison with values published by EUCAST and CLSI, as represented in Table 3.

**Table 3:** Resistance genes and antibiogram of carbapenemase positive *E*. *coli* and *K*. *pneumoniae* isolates.

Sample ID	Isolates	PCR	Carbapenems (Disc–μg)			Carbapenems (MIC–μg/ml)		
			MEM	IMI	ETP	MEM	IMI	ETP
001	*E*. *coli*	*blaOXA-48*	R	R	R	0.5 (S)	0.75 (S)	3.0 (R)
006	*K*. *pneumoniae*	*blaNDM*	R	S	R	1.0 (S)	1.0 (S)	2.0 (R)
061	*E*.*coli*	–	R	R	R	32 (R)	32 (R)	32 (R)
069	*K*. *pneumoniae*	*blaOXA-48*	R	R	R	0.25 (S)	0.75 (S)	1.0 (I)
100	*K*. *pneumoniae*	*blaOXA-48*	R	R	R	0.19 (S)	0.25 (S)	0.5 (S)
102	*E*. *coli*	–	R	S	R	0.094 (S)	0.25 (S)	0.38 (S)
103	*K*. *pneumoniae*	*blaOXA-48*	R	S	R	0.5 (S)	0.75 (S)	0.38 (S)
104	*E*. *coli*	–	R	S	R	0.25 (S)	2.0 (R)	0.25 (S)

MEM = Meropenem; IMI = imipenem; ETP = Ertapenem; R = Resistant; S = Sensitive; I = Intermediate; Values of MICs are presented with their susceptibility or resistance interpretations as n (R/S/I), – = Negative for the screened carbapenemase genes

### Phenotypic confirmation of carbapenemase production and distribution of carbapenemase-encoding genes among the *E*. *coli* and *K*. *pneumoniae* isolates

Concerning the phenotypic confirmation of carbapenem resistance, 75% (6/8) of the isolates that were resistant to carbapenems by disc diffusion were positive by the MHT and 75% (6/8) by the mCIM. Five out of the eight isolates (62.5%) exhibited carbapenemase activities by both methods. There was an equal distribution (3 *E*. *coli* and 3 *K*. *pneumoniae*) of positive tests by both MHT and mCIM among the resistant isolates.

The overall prevalence of carbapenemase genes by PCR was 62.5% (5/8), 80.0% (4/5) of which was accounted for by *blaOXA*-48 and 20.0% (1/5) by *blaNDM*. All the four *K*. *pneumoniae* isolates that were resistant to the carbapenems tested harboured 3 *blaOXA*-48 (*n* = 3) and *blaNDM* (*n* = 1); *blaOXA*-48 was detected in one *E*. *coli* isolate.

Both the MHT and the mCIM detected carbapenem resistance trait mediated by the *blaOXA*-48 gene, but not *blaNDM*. The study did not detect any of the *blaVIM*, *blaKPC*, and *blaIMP* genes. There were no bacterial isolates that harboured multiple carbapenem resistance genes. The age groups whose infecting isolates harboured carbapenemase genes were 10 years and below (40%, *n* = 2), 41 to 50 years (20%, *n* = 1), and above 60 years (40%, *n* = 2). Also, 3.5% (*n* = 3) of females and 3.4% (*n* = 2) of males were infected with carbapenemase gene-harbouring isolates. Table 4 presents a summary of the results on carbapenemase gene detection/expression.

**Table 4:** Summary of Results on the phenotypic and genotypic detection of carbapenem resistance.

Isolates	Phenotype			Genotype					
	Disc	MHT	mCIM	PCR	*blaOXA-48*	*blaNDM*	*blaKPC*	*blaVIM*	*blaIMP*
	*n* = 144	*n* = 8	*n* = 8	*n* = 8					
***K*. *pneumoniae***	4 (2.8)	3 (37.5)	3 (37.5)	4 (50.0)	3 (37.5)	1 (12.5)	–	–	–
***E*. *coli***	4 (2.8)	3 (37.5)	3 (37.5)	1 (12.5)	1 (12.5)	–	–	–	–
**Total**	8 (5.6)	6 (75)	6 (75)	5 (62.5)	4 (50)	1 (12.5)	–	–	–

Values are represented as *n* (%); PCR = polymerase chain reaction; DISC = disc diffusion method; MHT = modified Hodge test; mCIM = modified carbapenem inactivation method; Common carbapenemase genes = *blaOXA-48*, *blaNDM*, *blaKPC*, *blaVIM*, *blaIMP*; – = No gene detected

## Discussion

This study evaluated the prevalence of carbapenem resistance, AMR patterns, and the distribution of carbapenem resistance genes among MDR *E*. *coli* and *K*. *pneumoniae* isolated from clinical specimens in Accra, Ghana. It is one of the very few studies on carbapenem resistance in the country. The prevalence of carbapenem resistance (5.6%) seems higher than that reported in the recent study conducted in Ghana by Codjoe *et al*. [33] among Gram-negative bacilli– 2.9%–the majority of which were *Pseudomonas aeruginosa* and *Acinetobacter baumannii*. This is understandable, as the current study focused only on MDR isolates, while that of Codjoe *et al*. [33] did not restrict their focus to MDR isolates. Hackman *et al*. [32] and Owusu-Oduro [31] reported somewhat higher prevalence—7.2% and 10% respectively—but Hackman *et al*. [32] limited their study to CRE among phenotypic ESBL producers, and Owusu-Oduro’s [31] study was on Enterobacterales involved in UTI infection. The disparities between the CRE prevalence observed in the current study compared to these studies may be accounted for by the fact that the current study concentrated on MDR *E*. *coli* and *K*. *pneumoniae*, and these were isolated from 15 different types of samples. Regardless of the differences in prevalence, the studies suggest that carbapenem resistance is gradually emerging in Ghana. The prevalence recorded in the current study is consistent with those reported in some other African countries, such as Senegal (5.1%) [41], Nigeria (6.5%) [42], and South Africa (2.6–8.9%) [43]. Meanwhile, some other studies in Africa have reported a higher prevalence of CRE, such as 18.4% in Uganda [44] and 54.1% in Egypt [45]. Globally, there have been varying reports on the prevalence of CRE– 9.1% in Nepal [46], 5.74% in Malaysia [47], and 4.9% in Argentina [48]. These reports are indicative of a rise in carbapenem resistance worldwide, and measures need to be implemented urgently to curb this global menace.

The MDR isolates demonstrated high resistance rates to most of the antimicrobials tested, similar to patterns reported by Feglo & Adu-Sarkodie [49], Obeng-Nkrumah *et al*. [50], and Quansah *et al*. [51] in Ghana and Ranjbar *et al*. [52] in Iran. The high level of ampicillin resistance (100%) observed in *K*. *pneumoniae* might be attributed to the intrinsic resistance of *K*. *pneumoniae* to ampicillin because of the presence of SHV-1 beta-lactamase present on their chromosomes or transferable plasmids [53]. Interestingly, high resistance to commonly used antibiotics, such as penicillins and cephalosporins were recorded in this study; these drugs are part of the approved list of drugs for the treatment of bacterial infections in Ghana. It is, nonetheless, understandable that such rates were observed, as all the isolates investigated in the current study are known MDRs. Yet, the cause for concern cannot be ruled out, as these high rates were recorded against several of the antimicrobials. This observation adds to the several lines of evidence that support the need to strengthen antimicrobial stewardship programmes in Ghana and elsewhere [54–56]. Considering the resistance rates recorded against the carbapenems—ertapenem, meropenem, and imipenem—which in conjunction with the reports of Owusu-Oduro [31], Hackman *et al*. [32], Agyepong *et al*. [8], Codjoe *et al*. [33], and Quansah *et al*. [51] cited above, demonstrate the emergence of carbapenem resistance in the country, it is surprising that carbapenems, which are antibiotics of last resort for treating serious bacterial infections, are not included in the Ministry of Health’s Essential medicine list in Ghana [57]. This study provides further evidence that warrants the inclusion of carbapenems in the essential medicine list in the country.

All the eight carbapenem-resistant isolates showed 100% resistance to both meropenem and ertapenem, with 50% resistance recorded in imipenem. This high resistance to meropenem was also reported by Codjoe [35] in five different carbapenem-resistant isolates, including *K*. *pneumoniae*. Additionally, it has been reported that the disc diffusion test by ertapenem is a sensitive indicator of carbapenemase production [58], just as observed in this study. Understanding CPE antibiotic susceptibilities is critical for making therapy decisions [59], and from the E-test results of the current study, ertapenem MIC seemed to be the best predictor of carbapenemase producers. This finding is supported by Nordmann & Poirel [60], who reported that because ertapenem MIC values are typically greater than those of other carbapenems, it appears to be a promising choice for detecting most carbapenemase producers. However, Hara *et al*. [58] reported that CPE does not always show a minimum inhibitory concentration (MIC) value for carbapenems in the resistance range as seen in this study, making identification usually a problem. Hence detection of carbapenemase based on MIC reports alone may not be reliable, and other laboratory tests, such as molecular techniques, should be adopted.

Additionally in this study, the prevalence of carbapenem resistance according to the phenotypic confirmatory tests was found to be 75% (6/8) by both the MHT and mCIM. The study by Codjoe *et al*. [33] in Ghana showed a much lower prevalence of 18.9% by MHT, and may be due to most of their isolates being *Acinetobacter spp*. and *Pseudomonas spp*., in contrast with the current study that focused on *E*. *coli* and *K*. *pneumoniae*. Furthermore, of the three *E*. *coli* isolates that tested positive for MHT, two turned out to be false positives according to the PCR technique used in this study. This occurrence might be due to other mechanisms of resistance that might mimic carbapenemase activities, such as porins loss co-expressing with ESBL and AmpC [6, 33]. It could also be because in the current study, screening for carbapenemase genes focused only on commonly encountered carbapenemases, and not the less-frequently encountered ones. In contrast to findings from this study, another study by Quansah *et al*. [51], also in Ghana, showed that none of their carbapenem-resistant *Klebsiella spp*. tested positive for MHT, making MHT a less sensitive test for the detection of carbapenemase activity. Meanwhile, the results of this study prove MHT to be a valuable test for detecting carbapenemase activity. To the best of our knowledge, there are no published data in Ghana concerning the use of mCIM for the detection of carbapenemase activity. This might be because mCIM is a relatively new method, and the detection of carbapenemase by both phenotypic and genotypic methods is not incorporated into the routine microbiological practice in the various hospital laboratories in Ghana.

Among the eight carbapenem-resistant isolates, 50.0% harboured *blaOXA-48* and 12.5% *blaNDM* genes. In contrast, Quansah *et al*. [51] reported 2.16% and 0.72% for *blaOXA-48* and *blaNDM* respectively. Meanwhile, just as reported from this study, the predominant carbapenemase genes were *blaOXA-48* in *K*. *pneumoniae*. In addition, Codjoe *et al*. [33] also reported that 14.5% of their carbapenemase genes were *blaNDM*, which is similar to the proportion observed in this study (12.5%). The only *blaNDM* gene was identified in *K*. *pneumoniae*, and this is in agreement with a study by Hara *et al*. [58], who reported that the majority of Metallo beta-lactamase (MBL) producers are hospital-acquired and MDR *K*. *pneumoniae*. Additionally, Codjoe *et al*. [33], cited earlier, also reported on *blaOXA-48*, *blaNDM*, and *blaVIM* in their study. Though they reported *blaNDM* as the predominant carbapenemase gene in their study, all the *blaNDM* genes were mainly from *Acinetobacter* species (9 *blaNDM*-1), and less frequently *Pseudomonas aeruginosa* (2- *blaNDM*). The *E*. *coli* and *K*. *pneumoniae* from their study had 3 *blaNDM* and 2 *blaOXA-48*, respectively. In contrast, the *blaNDM* gene detected in this study was from *K*. *pneumoniae*. This shift might be due to ongoing interspecies transmission of resistance genes from *E*. *coli* to *K*. *pneumoniae* through transmissible plasmids that carry the *blaNDM* genes [61]. Hence this study, together with other studies in Ghana, seem to emphasize that the circulating carbapenemase genes among *E*. *coli* and *K*. *pneumoniae* in Ghana are predominantly *blaOXA-48* and *blaNDM*.

## Conclusion

It is concluded that although the rates of resistance demonstrated by the isolates against most of the antibiotics tested were high, the prevalence of carbapenemase producers was low. There is evidence that *blaOXA-48* and *blaNDM* may be circulating in Ghana, with *blaOXA-48* predominantly found in *K*. *pneumoniae* isolates.

The high resistance rates observed among the study isolates warrant a review of the approved list of drugs for the treatment of bacterial infections in Ghana. Moreover, the findings warrant an upscaling of AMR surveillance programmes and a fortification of infection prevention and control programmes in the country. Furthermore, genomic sequencing of the isolates, particularly the carbapenemase producers, is recommended to determine their lineage.
